# Oral amelanotic melanoma in a 73-year-old patient: A rare case report and literature review

**DOI:** 10.4317/jced.63521

**Published:** 2025-12-30

**Authors:** José-Alcides-Almeida de Arruda, Victor-Zanetti Drumond, Lucas-Guimarães Abreu, Cinthia-Verónica-Bardález-López de Cáceres, Pablo-Agustin Vargas, Patrícia-Carlos Caldeira, Felipe-Paiva Fonseca, Ricardo-Alves Mesquita, Bruno-Augusto-Benevenuto de Andrade, Tarcília-Aparecida Silva

**Affiliations:** 1 Department of Oral Diagnosis and Pathology, School of Dentistry, Universidade Federal do Rio de Janeiro, Rio de Janeiro, Brazil; 2 Department of Oral Surgery, Pathology and Clinical Dentistry, School of Dentistry, Universidade Federal de Minas Gerais, Belo Horizonte, Brazil; 3 Department of Child and Adolescent Oral Health, School of Dentistry, Universidade Federal de Minas Gerais, Belo Horizonte, Brazil; 4 Department of Oral Diagnosis, Piracicaba Dental School, Universidade Estadual de Campinas, Piracicaba, Brazil

## Abstract

Oral melanoma is an aggressive neoplasm that predominantly affects older adults. The amelanotic subtype is exceedingly rare, and its diagnosis is challenging due to the absence of pigmentation. Herein, we report a rare case of oral amelanotic melanoma in an older adult, discussed in light of the available literature. A 73-year-old Brazilian female patient presented with pain and discomfort in the left transverse palatal rugae. Clinically, the lesion appeared as a single, sessile, dome-shaped nodule with a coloration similar to the adjacent mucosa. Histopathological examination revealed a spindle-cell malignant neoplasm with epithelioid nests within a fibrous stroma, lacking melanin pigmentation. Immunohistochemistry was positive for pan-cytokeratin AE1/AE3, vimentin, S100 protein, Melan-A, SOX10, NSE, and TRP-2, with a high Ki-67 index. The patient died two months after diagnosis. A review of PubMed, Scopus, Embase, and Web of Science identified 35 cases of oral amelanotic melanoma in older adults, predominantly females (n=20/57.1%) in their 60s (n=16/45.7%). This report documents the sixth case of oral amelanotic melanoma in older adults from Latin America. Clinically, the case was challenging because it mimicked a non-neoplastic proliferative process and, microscopically, presented as an amelanotic variant. An immunohistochemical panel is recommended to avoid diagnostic pitfalls; this is the first report of TRP-2 immunoexpression in oral amelanotic melanoma.

## Introduction

Oral melanoma is an uncommon and highly aggressive malignant neoplasm originating from melanocytes within the ectoderm-derived mucosal epithelium (1 , 2). Epidemiologically, it accounts for 0.2%-0.8% of all melanomas, 31%-35.6% of mucosal melanomas, and approximately 0.03% of all malignant neoplasms (2 - 7). Historically, dark tumors described as melanosis were first reported as early as the 5th century B.C. by Hippocrates and later by Rufus of Ephesus. The first surgical description of melanoma is attributed to John Hunter in 1787, while William Norris provided the earliest account of amelanotic melanoma in 1857 (8). Amelanotic melanoma is a rare hypomelanotic variant, accounting for approximately 8% of all melanomas, with the head and neck affected in nearly 26% of cases (9 , 10). Oral amelanotic melanoma is exceedingly rare. In general, middle-aged men in their 50s, particularly with lesions involving the maxilla, are most frequently affected (11). At the molecular level, amelanotic melanoma is characterized by dysregulation of melanin synthesis, cell cycle regulation, and apoptotic pathways. Although tumor cells retain their melanocytic lineage and the inherent biological capacity to produce melanin, this ability is often absent or markedly diminished (12). Clinically, oral amelanotic melanoma is frequently misdiagnosed or detected at a late stage because it emulates non-neoplastic proliferative processes (11 , 13). For this reason, it has been termed "the great masquerader" (14). Histopathologically, most cases exhibit an epithelioid morphology, although spindle cell and desmoplastic patterns have also been described (12). Pigmentation is demonstrable microscopically in fewer than 5% of tumor cells yet remains clinically inapparent, further complicating diagnosis (15). Compared with conventional melanoma, oral amelanotic melanoma demonstrates greater aggressiveness, higher recurrence rates, and significantly worse survival outcomes (9). Therefore, incorporating oral melanoma screening into routine geriatric dental care, supported by structured diagnostic algorithms, may substantially improve early detection and survival in this vulnerable population (2). Given the scarcity of reports on oral amelanotic melanoma, particularly among older adults, we present to literature an additional case involving a 73-year-old female patient. We also provide a review of the available data on oral amelanotic melanoma in the geriatric population (60 years).

## Case Report

**Figure 1:**
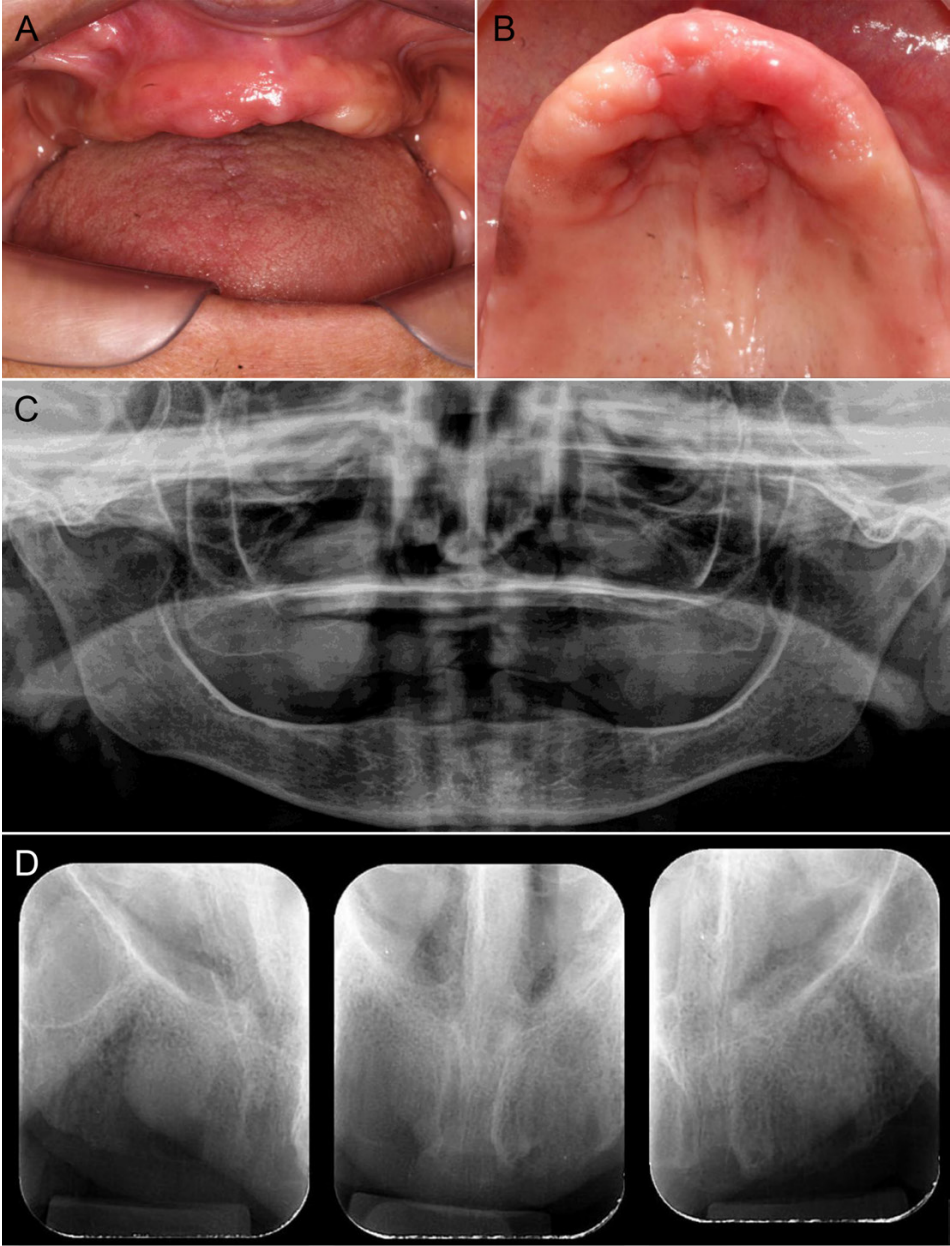
Clinical and radiographic features of oral amelanotic melanoma. (A) Intraoral view showing a completely edentulous maxilla. (B) Occlusal view of the maxilla revealing a single sessile, dome-shaped lesion located on the left transverse palatal rugae, measuring approximately 15×15×8 mm. The lesion exhibits a smooth surface and coloration similar to the adjacent mucosa. Discrete erythematous areas are observed on the anterior alveolar ridge, while the posterior and lateral ridges display multiple diffuse brownish macules of varying sizes. (C) Panoramic and (D) periapical radiographs showing no evidence of bone alterations.

A 73-year-old female Brazilian patient was referred to the oral medicine service due to pain and discomfort while wearing a maxillary complete denture. The duration of the symptom was unknown. She reported no history of smoking, alcohol consumption, or trauma to the maxillofacial region. Her family history was notable for type II diabetes and unspecified cancer-related deaths. Her medical history included type II diabetes mellitus, systemic arterial hypertension, bilateral cataracts, and Alzheimer's disease. She was under pharmacological treatment with captopril (50 mg/day), hydrochlorothiazide (25 mg/day), metformin (500 mg twice daily), and donepezil (5 mg/day). Extraoral examination revealed no lymphadenopathy in the cervical lymph node chain. Intraorally, a single sessile, dome-shaped lesion was observed on the left transverse palatal rugae, measuring approximately 15×15×8 mm. The lesion had a smooth surface, firm consistency on palpation, and coloration similar to the adjacent mucosa, without signs of ulceration. On the anterior alveolar ridge, discrete erythematous areas were noted, whereas on the posterior alveolar ridge, multiple diffuse brownish macules of varying sizes were in place. These pigmented lesions were flat (macular) with irregular contours, smooth surfaces, and no evidence of ulceration erosion or induration (Fig. 1A,B).

1

**Figure 2:**
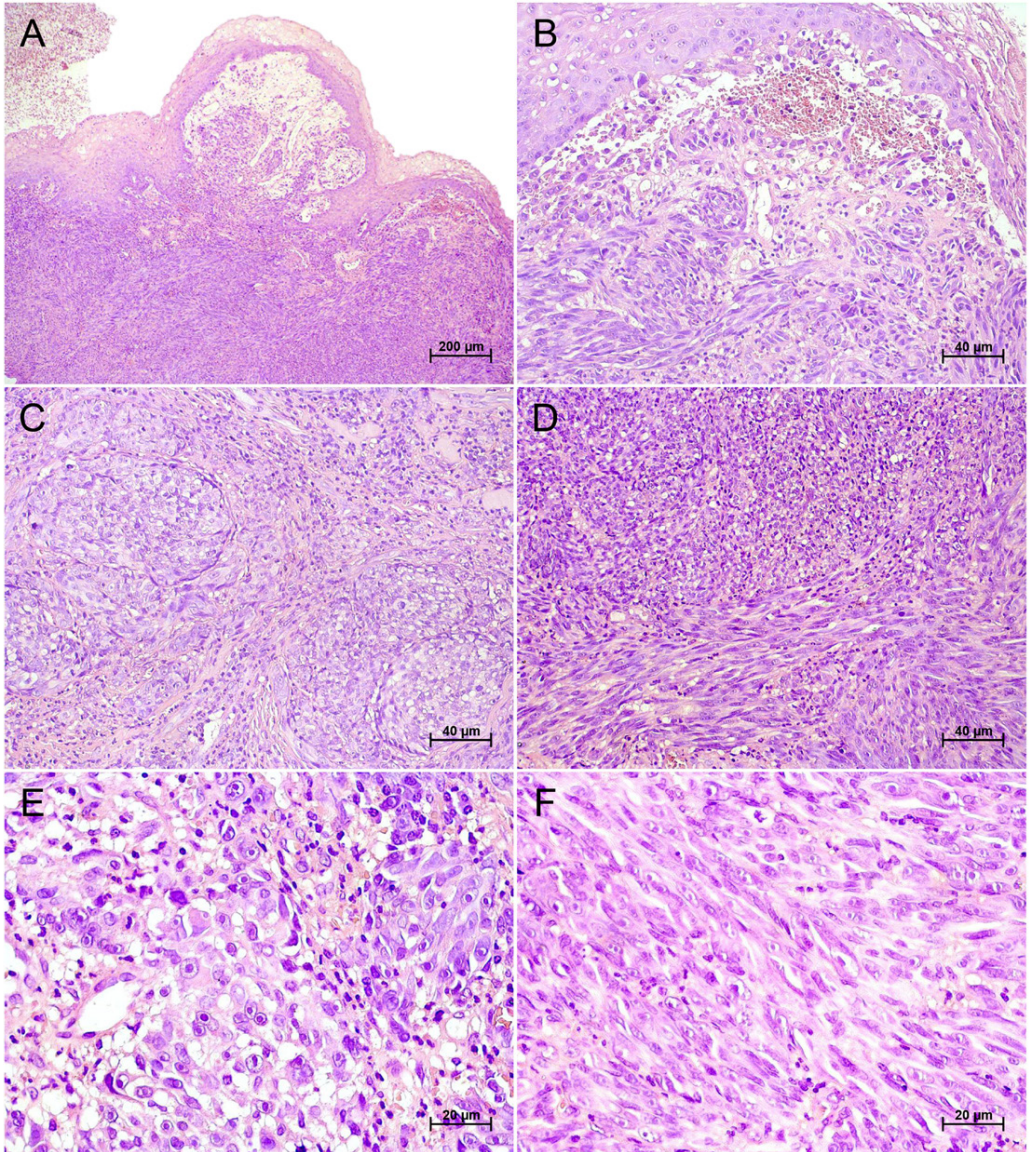
Histopathological features of oral amelanotic melanoma. (A) Low-power view of an exophytic, nodular, non-pigmented mucosal lesion composed of an infiltrative malignant neoplasm extending from the epithelial–connective interface into the underlying lamina propria. (B) Interface region showing atypical epithelioid melanocytic cells arranged in nests immediately beneath the surface epithelium. (C) Tumor nests of atypical epithelioid cells with enlarged nuclei, prominent nucleoli, and moderate nuclear pleomorphism. (D) Solid sheets of neoplastic cells with high cellularity embedded in a desmoplastic stroma. (E) High-power view of epithelioid tumor cells with vesicular nuclei, amphophilic nucleoli, abundant eosinophilic cytoplasm, frequent mitotic figures (including atypical forms), and absence of melanin pigmentation. (F) Spindle-cell component composed of elongated nuclei, hyperchromasia, and fascicular arrangement (hematoxylin and eosin staining; 40×, 200×, and 400×).

Panoramic and periapical radiographs revealed no evidence of underlying bone alterations (Fig. 1C,D). Based on the clinical characteristics, the primary diagnostic hypothesis for the dome-shaped lesion was inflammatory fibrous hyperplasia, whereas the differential diagnoses for the pigmented lesions included racial (physiological) pigmentation, drug-induced pigmentation, and post-traumatic or post-inflammatory hyperpigmentation. Blood tests revealed an HbA1c level of 8%, while the complete blood count and coagulation profile showed no significant abnormalities. Histopathological examination of the biopsy of the dome-shaped lesion revealed a malignant neoplasm composed predominantly of pleomorphic spindle cells arranged in interlacing fascicles, interspersed with ovoid and cuboidal epithelioid cells forming nests and solid cords within a dense and well-vascularized fibrous stroma. Focal areas of mixed inflammatory infiltrate were present, and no melanin pigment was observed in the neoplastic cells (Fig. 2).

2

**Figure 3:**
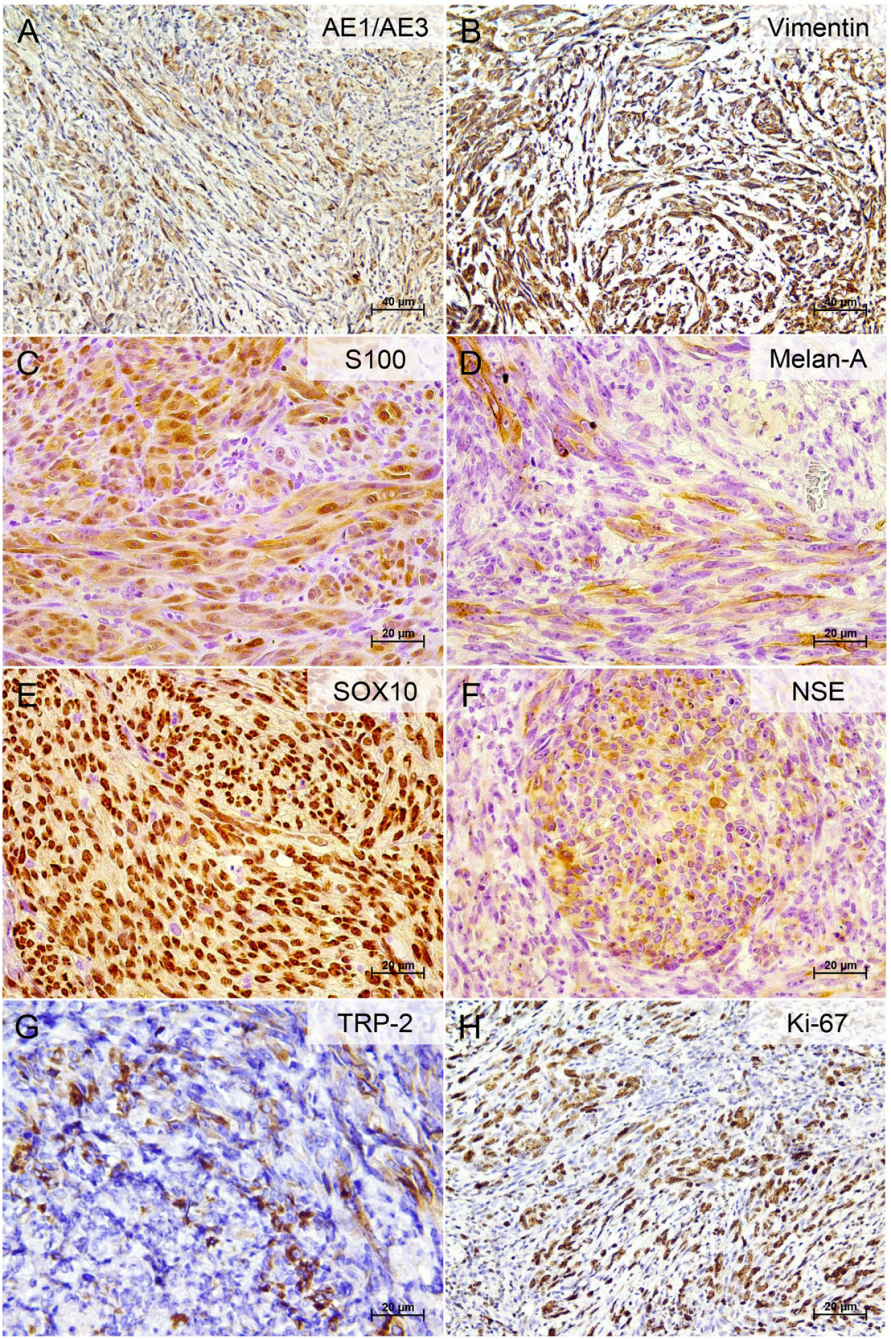
Immunohistochemical profile of the oral amelanotic melanoma. (A) Cytoplasmic immunoreactivity for pan-cytokeratin AE1/AE3 in spindle-shaped tumor cells (focal to moderate). (B) Strong cytoplasmic expression of vimentin in spindle cell areas. (C) Diffuse and intense nuclear and cytoplasmic positivity for S100 protein. (D) Focal cytoplasmic staining for Melan-A in tumor cells. (E) Strong and diffuse nuclear expression of SOX10 in tumor cells. (F) Cytoplasmic positivity for NSE in nodular aggregates of tumor cells. (G) Focal cytoplasmic expression of TRP-2 in scattered tumor cells. (H) Ki-67 nuclear staining indicating a proliferative index of 70% (immunohistochemistry with DAB (3,3′-diaminobenzidine) chromogen; 200× and 400×).

Immunohistochemically, the tumor cells exhibited strong immunopositivity for pan-cytokeratin (AE1/AE3), vimentin, S100 protein, Melan-A, SOX10, and NSE, as well as diffuse cytoplasmic staining for TRP-2. The Ki-67 labeling index was approximately 70% (Fig. 3).

3

Tumor cells were negative for HMB45, LCA, -SMA, and GFAP. Table 1 depicts information on the antibodies used. Based on these findings, a diagnosis of amelanotic melanoma was established. The patient was referred to an oncology service but died two months after the diagnosis without receiving any treatment.

Table 1

## Discussion

Data from a review in the electronic databases PubMed, Scopus, Embase, and Web of Science indicate that oral amelanotic melanoma in older adults is rare and has seldom been addressed in the literature, with only 35 cases documented hitherto (Table 2) (16 - 41).Table 2

Estimates of oral melanoma are largely derived from isolated case reports and small case series, with approximately 2,230 cases identified (1). This differs from oral amelanotic melanoma, for which only 55 cases have been reported thus far (11), and with cutaneous melanoma, which accounted for 325,000 new cases worldwide in 2020, with projections reaching 510,000 by 2040 (42). Previous research has indicated that 9% of oral melanoma cases correspond to the amelanotic variant (43). In the present review, Asia emerged as the leading continent (57.7%), with Japan accounting for 34.6% of all documented cases. It has been suggested that oral melanoma may occur more frequently in Asian populations, although the etiological basis remains elusive (1). In contrast, the relative proportion of oral melanoma among all mucosal melanomas in Caucasian populations is generally lower, although studies have reported comparable proportions of 30-50% in both Caucasian and Japanese cohorts (44 , 45). By comparison, the literature on cutaneous amelanotic melanoma is dominated by studies from Europe and North America (10). We herein contribute by describing a case of oral amelanotic melanoma in a 73-year-old patient, which likely represents the sixth reported case from Latin America.

Oral amelanotic melanoma shows a slight male predilection, similar to cutaneous forms, which affect men in approximately 55% of cases (9 , 11). Interestingly, our review demonstrated a female predominance (57.1%). Although sex distribution varies across studies (2), a systematic review reported a global male-to-female ratio of 1.2:1 (1). Age patterns were also distinctive. Most cases reported in the literature have occurred in the seventh decade of life, whereas a recent Brazilian series described a mean age of 53.6 years (38). Overall, the mean age at diagnosis of oral melanoma lies between 65 and 69 years, with incidence peaking at 70-79 years (2). Given this age profile, geriatricians and geriatric dentists should be particularly vigilant when non-pigmented or asymptomatic lesions arise. Integrating oral melanoma screening into whole-body skin examinations within geriatric care pathways is, therefore, strongly recommended (2).

Clinically, oral amelanotic melanoma presents as an insidious neoplasm with a broad spectrum of morphological appearances that are often deceptive and largely responsible for delayed diagnosis (11). In older adults, the mean time to detection was approximately six months. The nodular pattern was the most prevalent (34.3%), most commonly arising in the maxillary gingiva, while ulceroproliferative (25.7%), sessile, pedunculated, or erythematous forms were also noted. These findings reflect the clinical spectrum described by Bansal et al. (11) and Soares et al. (38), in which ulcerated/reddish masses of the palate and gingiva predominated. Only four oral amelanotic melanomas were reported in other locations, including the buccal mucosa (31 , 35), tongue (38), and lip (34). Most lesions measured between 10 and 60 mm, a range comparable to that of conventional oral melanoma (2). Approximately one-third of older adults reported pain (31.4%), whereas asymptomatic lesions were equally common (34.3%), illustrating the silent nature of the tumor in its early stages. Furthermore, the reviewed literature indicates that most patients (70%) presented with lymphadenopathy at the time of diagnosis.

The present case posed considerable diagnostic challenges, as the lesion clinically resembled a non-neoplastic proliferative process, i.e., an inflammatory fibrous hyperplasia. This presentation is reminiscent of a case reported by Soares et al. (38), involving a 33-year-old female with a small sessile nodule on the incisive papilla that was initially misdiagnosed as a pyogenic granuloma. Indeed, the literature emphasizes that oral amelanotic melanoma may atypically present as pyogenic granuloma-like lesions, with gingival cases frequently assigned a presumptive diagnosis of reactive processes (13). Such clinical overlaps are not a mere eventuality; they directly contribute to diagnostic delays and reinforce the principle that all lesions with a benign clinical appearance should undergo microscopic examination.

In our patient, additional contiguous mucosal alterations were also observed, including discrete erythematous macules and diffuse brownish macules on the alveolar ridge. At that stage, our differential diagnoses included physiological pigmentation, drug-induced pigmentation, and post-traumatic or post-inflammatory pigmentation. These considerations were supported by reports attesting that, in the skin, hydrochlorothiazide has been associated with hyperpigmentation, as has donepezil (commonly prescribed for dementia due to Alzheimer's disease), although no cases involving the oral mucosa have been documented (46). Likewise, drug-induced lichenoid reactions in the oral cavity are most often associated with nonsteroidal anti-inflammatory drugs and antihypertensive agents, including -blockers, angiotensin-converting enzyme inhibitors, and diuretics (e.g., hydrochlorothiazide) (47). However, these lesions are typically indistinguishable from oral lichen planus, and drug-related pigmentation can only be confirmed when a temporal association with medication use is established, along with resolution following drug withdrawal and exclusion of other etiologies (48). Another important aspect of the present report was the patient's diagnosis of Alzheimer's disease. To our knowledge, no prior studies have described oral amelanotic melanoma in an older adult with this condition. Of particular note, it has been demonstrated that melanoma cells require amyloid beta (A), a polypeptide involved in Alzheimer's disease, for survival and growth within the brain parenchyma (49). Although the primary tumor in our case was located in the palate, the possibility of distant metastasis cannot be entirely ruled out.

Histopathologically, the present case exhibited a spindle-cell predominance, which is the most frequent pattern in oral amelanotic melanomas. Nonetheless, the literature also documents epithelioid, round, and undifferentiated morphologies (38). In the absence of melanin, diagnosis becomes particularly challenging due to the highly variable sarcomatoid-like presentation of these lesions. Oral amelanotic melanoma may mimic other malignant neoplasms, including epithelial and mesenchymal tumors, lymphomas, and sarcomas. Consequently, immunohistochemical markers such as S100 protein, HMB45, and Melan-A are essential for establishing the diagnosis. When confronted with non-pigmented spindle or epithelioid tumors, a minimal diagnostic panel including S100 protein, SOX10, Melan-A, HMB45, and TRP-2 is recommended. SOX10, in particular, has proven to be a highly sensitive and specific nuclear marker, surpassing S100 in specificity and aiding the recognition of spindle-cell and desmoplastic variants often negative for Melan-A or MiTF (50 , 51). Interestingly, HMB45 was negative in our case. Two previous reports (29 , 51) have also described HMB45-negative oral amelanotic melanomas. Although HMB45 is widely used, it is less sensitive than S100 protein or Melan-A, and negativity should not preclude the diagnosis, particularly in spindle-cell and desmoplastic variants. Moreover, HMB45 and Melan-A may yield false negatives in amelanotic or desmoplastic melanomas and false positives in perivascular epithelioid cell or adrenocortical tumors, requiring interpretative caution (50).

Additionally, we demonstrated immunoexpression of TRP-2 in the present case. TRP-2 (dopachrome tautomerase/DCT) is a melanocyte differentiation enzyme involved in melanin synthesis and has been evaluated in human melanomas with variable sensitivity. Itakura et al. (54) reported TRP-2 positivity in 83% of primary and 100% of metastatic melanomas, while most nevi were negative, supporting its specificity. Strobel et al. (55) confirmed TRP-2 expression in approximately 50% of human melanomas and emphasized its association with a differentiated phenotype. Compared with conventional melanocytic markers, TRP-2 tends to be retained in certain amelanotic or desmoplastic variants in which HMB-45 or Melan-A may be absent, reinforcing its complementary diagnostic value (54 , 55). The inclusion of TRP-2 within "MDX-style" cocktails, combining HMB45, MART-1, and tyrosinase, has been shown to improve detection accuracy in diagnostically challenging lesions, particularly when integrated with SOX10 (50 , 56). To the authors' knowledge, this represents the first published report of TRP-2 immunoexpression in a human oral amelanotic melanoma.

The Ki-67 proliferation index in the present case was approximately 70%, consistent with evidence that oral amelanotic melanomas display higher proliferative indices (mean 64%) compared with their pigmented counterparts (mean 31%) (38). Such findings support the hypothesis that amelanotic tumors may represent a less differentiated, or even dedifferentiated, phenotype of conventional melanoma, associated with increased biological aggressiveness. Similar results have been reported in older patients, among whom spindle-cell morphology, variable HMB45 expression, and aggressive clinical outcomes were consistently reported (23 , 24 , 32).

Previous studies have shown that mutations in KIT, NRAS, and BRAF V600 are uncommon in oral melanomas, with reported frequencies around 10% or less (57 , 58). Ichimura et al. (59) further confirmed the absence of BRAF V600 mutations and identified amplifications in RICTOR, CDK4, MDM2, KDR, and NF1 as more frequent molecular events in oral melanomas. The literature addressing oral amelanotic melanoma is particularly scarce; nonetheless, a rare non-canonical BRAF double substitution (T599I/V600K) has been described in an amelanotic melanoma with oral metastases, underscoring the molecular heterogeneity of this phenotype (60). In our case, however, molecular testing was not performed, which represents a limitation of this report.

Findings from the present review revealed a mortality rate of 94.4%. These results reinforce the poor prognosis of oral melanoma and the limited effectiveness of conventional therapies, consistent with previous reports highlighting surgery as the mainstay, but rarely curative approach (1). Radiotherapy and chemotherapy, when used in isolation, were almost uniformly associated with poor survival, in line with the intrinsic radioresistance and chemoresistance of mucosal melanomas (61). Even multimodal regimens combining surgery, radiotherapy, and/or chemotherapy frequently resulted in disease progression, recurrence, or distant metastases. Immune checkpoint blockade and molecularly targeted therapies have shown promise in cutaneous melanoma and selected mucosal subtypes but remain underexplored in oral presentations (62). It is important to note that older adults with oral melanoma often face additional challenges related to treatment tolerability. Diagnostic delays further contribute to high mortality rates, as most cases are identified at stage III or IV, frequently with nodal or distant metastases (1). Survival rates for oral amelanotic melanoma are considerably lower than those for conventional oral melanoma, with 3-year and 5-year survival rates of 18.75% and 6.25%, respectively (11). We have previously emphasized the importance of a multidisciplinary team in delivering supportive care, addressing both physical and psychosocial needs, and ensuring comfort and dignity during the terminal phase of the disease (2).

In summary, this report describes the sixth documented case of oral amelanotic melanoma in an older adult from Latin America. Given its rarity and aggressive behavior, diagnosis is particularly challenging, as it often mimics non-neoplastic or reactive proliferations in clinical presentations. In the absence of melanin, a panel of immunohistochemical markers is indispensable to confirm the diagnosis.

## Figures and Tables

**Table 1:** Antibodies employed in the immunohistochemical analysis of the reported case.

Antibodies	Clone	Supplier	Nature of antibodies	Dilution
LCA	2B11 + PD7/26	Dako	Monoclonal (mouse)	Ready to use
AE1/AE3	AE1/AE3	Dako	Monoclonal (mouse)	Ready to use
α±-SMA	1A4	Dako	Monoclonal (mouse)	1:50
Vimentin	EP21	Cell Marque™	Monoclonal (mouse)	1:200
S100	EP184	Cell Marque™	Polyclonal (rabbit)	1:100
HMB45	HMB45	Cell Marque™	Monoclonal (mouse)	1:100
Melan-A	A103	Sigma-Aldrich	Monoclonal (mouse)	1:200
SOX10	EP268	Cell Marque™	Monoclonal (mouse)	1:20
NSE	MRQ-55	Cell Marque™	Monoclonal (mouse)	1:200
TRP-2	EPR21986	Abcam	Polyclonal (rabbit)	1:1000
GFAP	6F2	Dako	Polyclonal (rabbit)	1:100
Ki-67	MIB1	Dako	Monoclonal (mouse)	1:100

Note: AE1/AE3, pan-cytokeratin; GFAP, Glial Fibrillary Acidic Protein; HMB45, Human Melanoma Black 45; LCA, Leukocyte Common Antigen; Melan-A, Melanoma Antigen Recognized by T Cells (MART-1/Melan-A); NSE, Neuron-Specific Enolase; SOX10, SRY-Box Transcription Factor 10; TRP-2, Tyrosinase-Related Protein 2; α-SMA, Alpha-Smooth Muscle Actin.

**Table 2:** Clinicodemographic, histopathological, and immunophenotypic characteristics of older adults (≥60 years) with oral amelanotic melanoma, based on case reports and case series available in PubMed, Scopus, Embase, and Web of Science databases*.

Study	Age/sex	Clinical and radiographic features	Evolution (mo)	Histopathological features	Lymph node involvement	IHC markers	Treatment, recurrence, and metastasis	Follow-up (mo)	Outcome
Takahashi & Seiji, 1974; Japan [16]	74/F	Asymptomatic nodule in the maxillary gingival region	1	Spindle cell and presence of melanin	Yes	NR	NR	NR	NR
Chu et al., 1993; Japan [17]	79/F	Painful nodule in the maxillary gingival region, measuring 52×35×21 mm, with associated bone destruction	1	Round spindle cells	Yes	S100+	Surgical excision	NR	NR
Tani et al., 1994; Japan [18]	62/M	Painful nodule in the maxillary gingival region	0.5	NR	NR	NR	Chemotherapy and immunotherapy and distant metastasis to the lung	NR	NR
Kimijima et al., 1999; Japan [19]	84/F	Asymptomatic pedunculated lesion with associated bone destruction	0.5	Spindle cell and presence of melanin	Yes	HMB45+	Radiotherapy; recurrence in nasal cavity and ethmoidal sinuses after 13 months	24	Deceased
Ohno et al., 2000; Japan [20]	70/M	Painful, bleeding nodular lesion in the maxillary gingival region, measuring 48x40 mm, with associated bone destruction	3	Undifferentiated cells	Yes	HMB45+, S100-, AE1/AE3-, and vimentin-	Radiotherapy and chemotherapy; recurrence in orbital region and maxillary sinus; distant metastasis to the lung	12	Deceased
Kao et al., 2001; Taiwan [21]	80/M	Painful, erythematous, irregular lesion in the hard palate, measuring 40×50 mm	6	Spindle cells and absence of melanin	NR	HMB45+, S100+, and AE1/AE3+	NR	NR	NR
Ducic & Pulsipher, 2001; USA [22]	78/F	Erythematous lesion on the soft palate, measuring 20 mm	NR	Spindle cells and presence of melanin	Yes	HMB45+ and S100+	Surgical resection	12	NR
Notani et al., 2002; New Zealand [23]	78/F	Asymptomatic, pinkish-red, sessile ulcerated lesion on the hard palate, with associated bone destruction	4	Spindle cells and absence of melanin	Yes	HMB45+ and S100+	Surgical resection; multiple distant metastases	12	Deceased
	69/M	Asymptomatic, pinkish-red, sessile ulcerated lesion in the retromolar region, with pigmentation on the anterior palate and associated bone destruction	6	Epithelioid cells and absence of melanin	Yes	HMB45+ and S100+	Surgical resection and chemotherapy; distant metastases to the lung and liver	27	Alive
Tanaka et al., 2004; Japan [24]	82/F	Nodular lesion in the maxillary gingival region, measuring 45×46 mm	NR	Spindle cells	Yes	HMB45+ and S100+	Radiotherapy; distant metastases to the lung	NR	Deceased
	71/F	Nodular lesion in the maxillary gingival region, measuring 55×45 mm	NR	Spindle cells	Yes	HMB45+ and S100+	Chemotherapy, surgical resection, and immunotherapy; distant metastases to the heart	49	Deceased
	70/F	Nodular lesion in the maxillary gingival region, measuring 25×25 mm	NR	Spindle cells	No	HMB45+ and S100+	Surgical resection, chemotherapy, and immunotherapy; distant metastases to the lung	9	Deceased
	84/F	Nodular lesion in the maxillary gingival region, measuring 30×40 mm	NR	Spindle cells	No	HMB45+ and S100+	Surgical resection and radiotherapy	29	Deceased
	64/M	Nodular lesion in the hard palate, measuring 13×14 mm	NR	Spindle cells	No	HMB45+ and S100+	Surgical resection and radiotherapy	48	Deceased
	60/F	Nodular lesion in the maxillary gingival region, measuring 30×25 mm	NR	Spindle cells	No	HMB45+ and S100+	Radiotherapy; distant metastasis to the lung	75	Deceased
	71/M	Nodular lesion in the hard palate, measuring 38×11 mm	NR	Spindle cells	Yes	HMB45+ and S100+	Radiotherapy	12	Deceased
Cicconetti et al., 2009; Italy [25]	65/F	Painful, pedunculated, ulcerated lesion on the maxillary alveolar ridge, measuring 2×2×1 mm	24	Spindle cells	No	S100+ and AE1/AE3-	Surgical resection and radiotherapy	36	Deceased
Dominiak et al., 2011; Poland [26]	65/F	Asymptomatic, pinkish-red, ill-defined lesion on the hard palate, measuring 20 mm	0.5	Round cells	Yes	Melan-A+, HMB45+, and S100+	Surgical resection and radiotherapy; distant metastasis to the lung and liver	NR	NR
Kawasaki et al., 2011; Japan [27]	85/F	Asymptomatic, ill-defined lesion on the mandibular alveolar ridge, measuring 48×27 mm	0.5	Round cells	Yes	S100+, Melan-A+, HMB45+, vimentin+, AE1/AE3-, EMA-, and LCA-	Radiotherapy	NR	Deceased
Patil et al., 2011; India [28]	75/F	Painful, pinkish-red, sessile ulcerated lesion with associated bone destruction	2	Round cells	Yes	HMB45+, S100-, CK-, and LCA-	No treatment performed	NR	NR
Bansal et al., 2012; India [29]	75/F	Asymptomatic, ulcerated lesion on the mandibular alveolar ridge, measuring 3×3×2 mm, with associated bone destruction	2	Round cells	Yes	CK-, vimentin-, desmin-, HMB45-, Melan-A+, and S100+	No treatment performed	NR	NR
Jou et al., 2012; Brazil [30]	62/M	Painful, sessile lesion in the hard palate with a pigmented area in the mid-palatine region		Round to spindle cells	NR	S100+ and AE1/AE3-	Surgical resection and chemotherapy	NR	Deceased
Sakamoto et al., 2015; Japan [31]	97/M	Asymptomatic, pinkish-red nodular lesion on the buccal mucosa, maxillary gingiva, and tongue	1	Spindle cells	Yes	S100+ and HMB45+	Surgical excision	NR	NR
Ohnishi et al., 2015; Japan [32]	80/M	Painful, ill-defined erythematous lesion in the maxillary gingival region, measuring 40×50 mm, with associated bone destruction	1	Epithelioid cells and presence of melanin	Yes	S100+, HMB45+, and Melan-A+	Surgical excision and chemotherapy	NR	NR
de Paulo et al., 2015; Brazil [33]	65/M	Asymptomatic, sessile, ulcerated lesion on the hard palate, measuring 50×60 mm	NR	Epithelioid cells	Yes	AE1/AE3-, vimentin+, S100+, and Melan-A+	NR	NR	Deceased
Cooper et al., 2021; USA [34]	77/M	Pinkish-red lesion on the lip, measuring 12×10 mm	NR	NR	NR	S100+	NR	NR	NR
Nwoga et al., 2019; Nigeria [35]	62/M	Painful, grayish lesion on the hard palate	12	Absence of melanin	No	NR	No treatment performed	NR	NR
	61/F	Painful, reddish lesion on the buccal mucosa	36	NR	No	NR	NR	NR	NR
Raha et al., 2019; Iran [36]	61/F	Asymptomatic, pinkish-red lesion on the mandibular alveolar ridge, with associated bone destruction	NR	Round to spindle cells and presence of melanin	Yes	Melan-A+, S100+, and Ki67+	Surgical resection	NR	NR
Kim et al., 2020; Korea [37]	63/M	Painful, bleeding, well-circumscribed lesion in the mandibular gingival region, measuring 30×25 mm, with associated bone destruction	12	Spindle cells and absence of melanin	No	S100+, HMB45+, SMA-, and desmin-	Surgical resection	NR	NR
Soares et al., 2021; Brazil [38]	77/M	Asymptomatic, pinkish-red, ulcerated lesion in the anterior region of the tongue	NR	Epithelioid cells	Yes	Vimentin+, S100+, HMB45+, SOX10+, AE1/AE3-, LCA-, and α±-SMA-	Surgical resection and chemotherapy	11	Deceased
	68/M	Asymptomatic, pinkish-red, ulcerated lesion on the maxillary alveolar ridge	NR	Undifferentiated cells	No	Vimentin+, S100+, HMB45+, SOX10+, AE1/AE3-, LCA-, and α±-SMA-	NR	NR	NR
Rodrigues et al., 2021; Brazil [39]	64/F	Yellowish lesion in the mandibular gingival region, measuring 2.5 mm	NR	Absence of melanin	Yes	HM45+, S100+, and Melan-A+	Surgical resection and chemotherapy	9	Deceased
Panda et al., 2022; India [40]	60/F	Ulceroproliferative, irregular lesion on the hard palate, measuring 1.5×2 mm	4	Spindle and epithelioid cells	Yes	HMB45+, S100+, CD45-, desmin-, and AE1/AE3-	No treatment performed; distant metastasis to the brain	NR	Deceased
Lobekk et al., 2023; Norway [41]	60/F	Multifocal lesions: tumoral lesion in the maxillary gingival region, measuring 5×6 mm; ulcerated lesion in the hard palate; and diffuse pigmented lesion in the soft palate. The initial biopsy was performed in the non-pigmented gingival lesion	NR	Spindle cells organized in nests and absence of melanin	NR	HMB45+, S100+, SOX10+, and Melan-A+	Surgical resection and immunotherapy; recurrence	18	NR

Note: AE1/AE3, pan-cytokeratin; CK, cytokeratin; EMA, epithelial membrane antigen; F, female; HMB45, Human Melanoma Black 45; IHC, immunohistochemical; LCA, leukocyte common antigen; M, male; mo, months; NR, not reported; SOX10, SRY-box transcription factor 10; α-SMA: alpha smooth muscle actin. *A detailed description of the literature review procedure is provided in Supplementary File 1.

## Data Availability

The datasets used and/or analyzed during the current study are available from the corresponding author.
